# Wikidata for botanists: benefits of collaborating and sharing Linked Open Data

**DOI:** 10.1093/aob/mcaf062

**Published:** 2025-06-07

**Authors:** Sabine von Mering, Siobhan Leachman, Joaquim Santos, Heidi M Meudt

**Affiliations:** Museum für Naturkunde - Leibniz Institute for Evolution and Biodiversity Science, 10115 Berlin, Germany; Independent researcher, Wellington, 6021, New Zealand; Centre for Functional Ecology, Department of Life Sciences, University of Coimbra, 3000-456 Coimbra, Portugal; Museum of New Zealand Te Papa Tongarewa, Wellington, 6011, New Zealand

**Keywords:** Biodiversity data, Bionomia, botanists, collaboration, digital outreach, digital tools, herbaria, identifier, knowledge graph, Linked Open Data (LOD), open science, Wikidata

## Abstract

**Background:**

Wikidata is a multilingual linked open knowledge base to which anyone can contribute that contains multitudes of botany-related information. Wikidata reveals interactions between entities and connects botany-related information from multiple institutions and other sources, benefiting the botanical community in numerous ways. The aim of this article is to give an overview of Wikidata from a botany perspective and issue a call to action to the botanical community to collectively improve the quantity and quality of information related to botany, botanists and botanical collections, in Wikidata. Here, we use a broad definition of botany to include the study of many different taxa and specializations.

**Scope:**

Wikidata contains botany-related data and identifiers for botanists and botanical collectors, botanical taxa, natural history institutions and collections, botany-related publications, geographical locations and research expeditions, as well as genes, genetic variants, chemical compounds, diseases, and more. As an open, collaborative and community-curated knowledge base, Wikidata enables different communities to add and link data related to botany and empowers the querying and reuse of this data via digital tools such as the Wikidata Query Service, Bionomia, Scholia, TL-2 and Expeditia.

**Conclusions:**

Collaboration is key in botany and Wikidata, and the sharing and enriching of botany-related Linked Open Data benefits us all. Several resources, including ethical and legal guidelines, are available for botanists to edit, use, reuse, roundtrip and teach Wikidata. We call on all botanists to be active participants in Wikidata, improving the quality, quantity and linking of botany-related data. Our individual and collective actions can help harness the power of Linked Open Data to answer important queries in the field, improve accessibility of herbaria, increase visibility of botanists and their scientific contributions, integrate Wikidata into the classroom, support the Madrid Declaration strategic actions, achieve our collective goals, and ultimately make botany-related information more FAIR (Findable, Accessible, Interoperable, Reusable) and equitable.

## INTRODUCTION

Wikidata (Q2013) is a tool used to link data about many things. The following botanical story indicates how Wikidata can assist in providing context and enable the linking of botanical data.

The famous French botanist and explorer Auguste de Saint-Hilaire (Q707961) collected many specimens throughout his life. Between 1816 and 1821 he explored Brazil (Q155), where he collected a specimen from which he described a new species to science: *Oxalis insipida* A.St.-Hil. (Q39395060; https://www.gbif.org/occurrence/438235985). In 1832, Charles Darwin (Q1035), travelling aboard HMS *Beagle* (Q35926), stopped at the Island of Fernando de Noronha, Brazil (Q2438090). Among his collections was a specimen of *Oxalis* L. that was used many years later by Daniel Oliver (Q1161960) to publish a new species in William Jackson Hooker’s *Icones Plantarum* (Q5901658): *Oxalis noronhae* Oliv. (Q131343589; https://www.gbif.org/occurrence/4951949000). More than a century later, the Franco-Argentinian botanist Alicia Lourteig (Q454806) held this specimen in her hands and concluded that it was the same species as the Saint-Hilaire specimen, as we can see by her determination slip (Q131467875) from 1957. In fact, in her revision of the genus *Oxalis* (Q157378) (Lourteig, 1994), Lourteig reclassified this taxon as a subspecies of *Oxalis psoraleoides* Kunth (Q131350563), a species described by Carl Sigismund Kunth (Q77074) in 1821 in the journal *Denkschriften der Königlichen Akademie der Wissenschaften zu München* (Q20014480) based on a specimen collected by Alexander von Humboldt (Q6694) and Aimé Bonpland (Q405702) in Colombia (Q739) during their famous expedition to America between 1799 and 1804 (Q130639025; https://www.gbif.org/occurrence/694191712). More recently, phylogenetic studies have provided new insights into the evolution and relationships in this group. A recent molecular phylogenetic study of *Oxalis* subg. *Thamnoxys* (Endl.) Reiche (Q21382429) (which includes *O. psoraleoides* subsp. *insipida* (A.St.-Hil.) Lourteig, Q111323930) showed that the traditional taxonomic arrangement did not match genetics (Cabral *et al.*, 2024). It is worth mentioning that, following standard practice, all DNA sequences from that study are available in GenBank (Q901755) for others to use (National Library of Medicine, 2025). Searching the literature, one learns that the flowers of *O. psoraleoides* have been visited by both the hummingbird *Chlorostilbon lucidus* (Q1262955; Las-Casas *et al.*, 2012) and the honeybee *Apis mellifera* (Q30034; Nascimento *et al.*, 2014). The species is also used as food by three rural communities in the state of Pernambuco, Brazil (Q40942; Lucena *et al.*, 2007), and is cited as a traditional antimalarial (Q521616) in Peru (Q419; Milliken *et al.*, 2021).

**Fig. 1.**
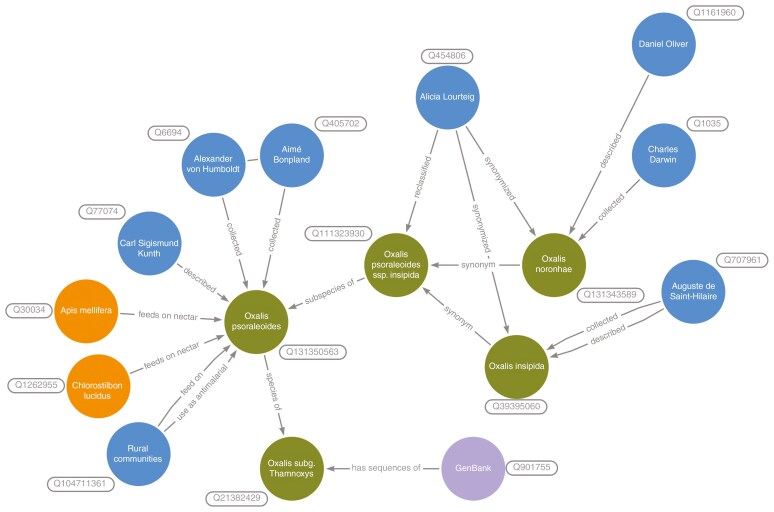
Knowledge graph of entities linked to *Oxalis psoraleoides* subsp. *insipida* (Q131350563) (A. St.-Hil.) Lourteig described in the above botanical story. Available online at https://commons.wikimedia.org/wiki/File:Fig_1_Oxalis_psoraleoides_ssp._insipida_Knowledge_Graph.jpg, Joaquim Santos, CC0 (https://creativecommons.org/publicdomain/zero/1.0/) via Wikimedia Commons.

This story illustrates what we can learn when we start pulling the thread that constitutes the network of knowledge. All these explorers, expeditions, botanists, scientists, species and places—in short, all these entities (Q35120)—are connected with each other (Fig. 1). Similarly, there is a huge amount of botany-related information that has been published over centuries that contains hidden connections between such entities. Much of this textual information is made available on the internet in a digital format. This information is usually unstructured, and hence is siloed, lacking in context, and not interoperable. In addition, information in different biodiversity databases as well as digital libraries is often not linked (e.g. Thomas, 2009; Page, 2022, 2023). Despite platforms, projects, initiatives and individuals attempting to rectify this problem (e.g. Plazi, Q7203726; Agosti and Egloff, 2009; Biodiversity Heritage Library, BHL, Q172266), there is still a long way to go.

Numerous platforms exist that publish botanical data and other botany-related information, including BHL, Global Biodiversity Information Facility (GBIF, Q1531570), Global Registry of Scientific Collections (GRSciColl, Q98594614), Index to Plant Chromosome Numbers (IPCN, Q131378293), International Plant Names Index (IPNI, Q922063), Palynological Database (PalDat, Q59786289), TRY Plant Trait Database (Q113391569), World Flora Online (WFO, Q77076820), and so on. However, their scope tends to be relatively specific. Furthermore, these platforms are not always integrated and can fail to include appropriately structured and searchable connections to broader yet relevant information. Such relevant information about botanists might include their employers and other career details, collaborators, geographical placements or kin relationships; about botany-related publications such as disambiguated authors, publishing journals or papers that have been cited in the publication; and about taxa such as publications giving the original description, images of the taxon, geographical range or taxon traits.

Wikidata can assist with tackling this issue of unstructured and unlinked data. By creating or enriching content on Wikidata, this knowledge base can be used to transform unstructured data into a structured format, with values assigned to predefined fields. Publishing information in Wikidata ensures it is findable, able to be accessed, interoperable (the structure follows a documented standard), and obstacle-free with regard to reuse (licensing), thus progressing towards achieving compliance with FAIR principles (Findability, Accessibility, Interoperability, and Reuse of digital assets, Q29032644; Wilkinson *et al.*, 2016). By empowering the botanical and broader communities to work together to collaboratively create Linked Open Data (LOD, Q18692990), Wikidata can transform siloed data into interoperable knowledge, linking and connecting these data and thus ensuring this knowledge is accessible and reusable.

**Fig. 2.**
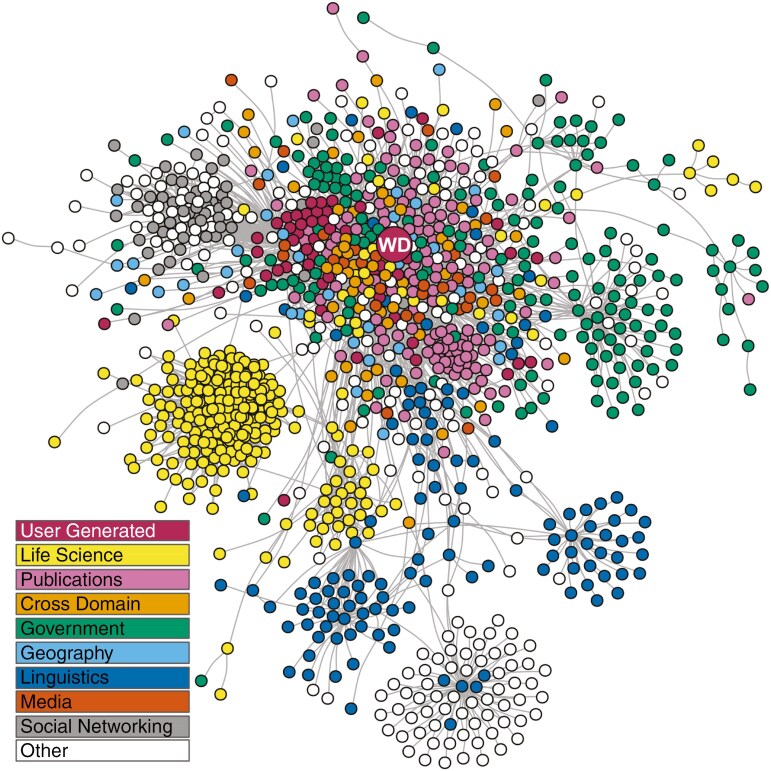
Wikidata in the Linked Open Data Cloud. Databases are indicated as circles (with Wikidata labelled as ‘WD’), with grey lines linking databases in the network if their data are linked. (Layout by graphopt algorithm in the igraph package in R. Data from https://lod-cloud.net/datasets. Text and image available online at https://commons.wikimedia.org/wiki/File:Wikidata_in_the_Linked_Open_Data_cloud.jpg, Thomas Shafee, CC BY 4.0 (https://creativecommons.org/licenses/by/4.0), via Wikimedia Commons. A searchable version of the same cloud can be found here: https://lod-cloud.net/clouds/lod-cloud.svg.

Once added to Wikidata, the information is transformed into Linked Open Data as the data are available in RDF dumps (Resource Description Framework; Berners-Lee, 2006; Erxleben *et al.*, 2014; Wikidata, 2025*a*; Fig. 2). This in turn opens a world of possibilities. It is worth mentioning that Wikidata was not built specifically for botany or any other field. Wikidata was originally designed to overcome the difficulties of accessing data in multiple-language Wikipedia articles and was intended to provide a central repository empowering access and the ability to reuse these data (Vrandečić *et al.*, 2023). The use of Wikidata has subsequently been adopted by multiple scientific and humanities disciplines (Waagmeester *et al.*, 2020, 2021; Lemus-Rojas *et al.*, 2022; Zhao, 2023; Shafee *et al.*, 2023; Kaiser and von Mering, 2024; Matos *et al.*, 2024; Turki *et al.*, 2024).

**Fig. 3.**
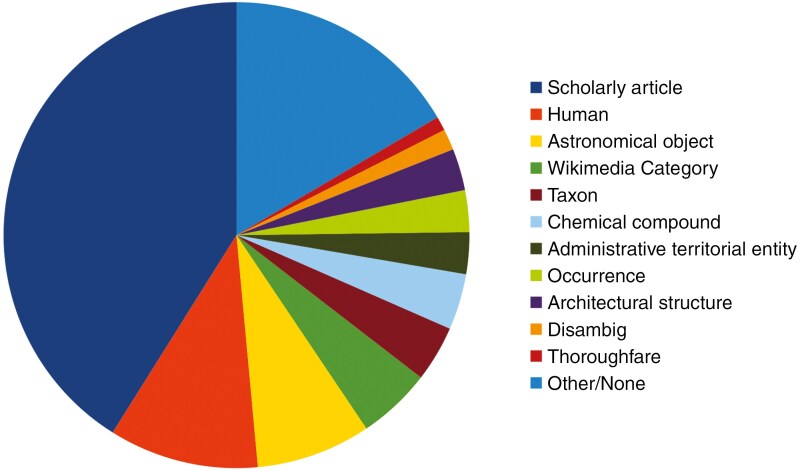
Visualization of all Wikidata content as at 8 April 2024, generated using data from QLever SPARQL queries for *instance of* (P31) and subclasses. Available online at: https://commons.wikimedia.org/wiki/File:Wikidata_content_2024.svg, VIGNERON, CC0 1.0 (https://creativecommons.org/publicdomain/zero/1.0/deed.en), via Wikimedia Commons.

Wikidata is a sister project to the well-known online encyclopaedia Wikipedia (Q52), and both are platforms of the Wikimedia Foundation. The thousands of individuals throughout the world who edit in this so-called Wikiverse (Q130313479) are called Wikimedians (Q41546637). Since its launch in 2012, Wikidata has been populated with a wide variety of data by individual Wikidata editors, who number ~25 000 worldwide (see https://www.wikidata.org/wiki/Wikidata:Statistics, date last accessed, 15 May 2025). Today, Wikidata comprises >100 million *items* from all fields of knowledge (Shafee *et al.*, 2023). Of these, >12 million are people (see Wikidata query https://w.wiki/CU2w, date last accessed 15 May 2025; Fig. 3), with 68 755 listed as having their *occupation* (P106) as botanist (Q2374149) (see Wikidata query https://w.wiki/CTZA, date last accessed, 15 May 2025). Similarly, there are currently about 4.1 million taxa (Fig. 3) including 3.2 million species in Wikidata (see Wikidata query https://w.wiki/CS4k, date last accessed 15 May 2025), and of these, for example, ~730 000 are angiosperm species names (https://w.wiki/CUZn).

**Fig. 4.**
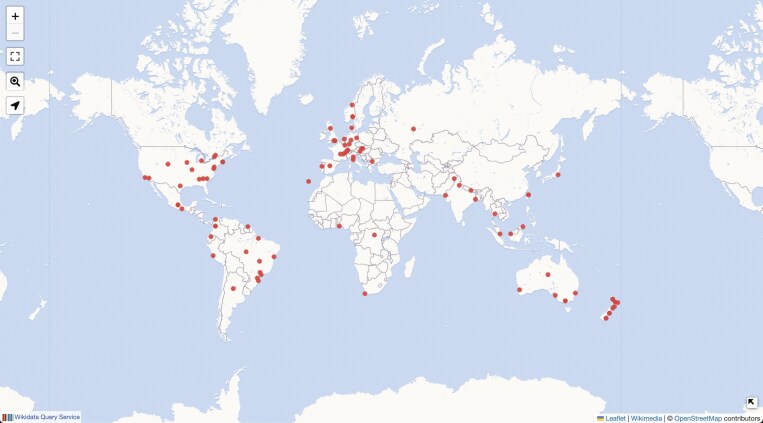
Visualization of global data for herbaria of the world, generated using a query in the Wikidata Query Service (https://w.wiki/CU4r). Note this query assumes that *items* for herbaria will have geographical coordinates on them, but as of December 2024 only 106 Wikidata *items* for herbaria have coordinates. The herbarium itself (not just its host institution) must have a Wikidata *item* with geographical coordinates to be included in this query. This visualization highlights not only the lack of separate Wikidata *items* for herbaria that include coordinates (with only ~3 % represented of a total of 3400; Thiers, 2025) but also a gap in Global South institutions. Both would be straightforward for the botanical community to fix by adding the missing information to Wikidata. Available online at: https://commons.wikimedia.org/wiki/File:Wikidata_query_herbaria_of_the_World.jpg, Wikimedia Foundation, CC BY-SA 4.0 (https://creativecommons.org/licenses/by-sa/4.0/deed.en), via Wikimedia Commons.

**Fig. 5.**
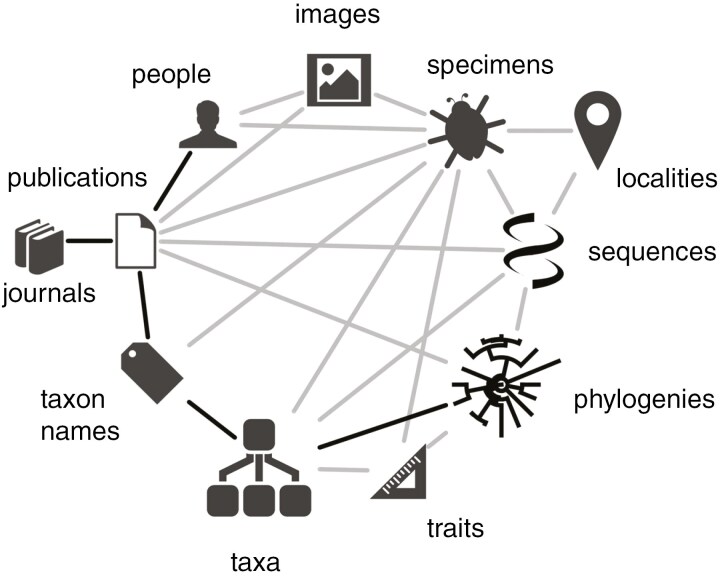
Biodiversity knowledge graph from Page (2013) showing the relationships (as grey or black lines) between botanists, publications, plant taxa and other botany-related data. Available online at https://commons.wikimedia.org/wiki/File:Biodiversity_knowledge_graph_by_Rod_Page.jpg, Rod Page, CC BY 4.0 (https://creativecommons.org/licenses/by/4.0), via Wikimedia Commons.

With the structured botany-related data already available in Wikidata, which itself is only a fraction of the total potential data that can be linked in Wikidata, it is possible to reveal interactions that can help expand botany-related knowledge. This expansion would be very difficult to achieve without the help of tools that query and display such data (Figs 2–4). Botany-related data in Wikidata can also feed into the biodiversity knowledge graph (Waagmeester *et al.*, 2020; Page, 2022), a network of linked biodiversity data that visualizes the digital linking possible between different biodiversity-related entities (Page, 2013; Fig. 5).

**Fig. 6.**
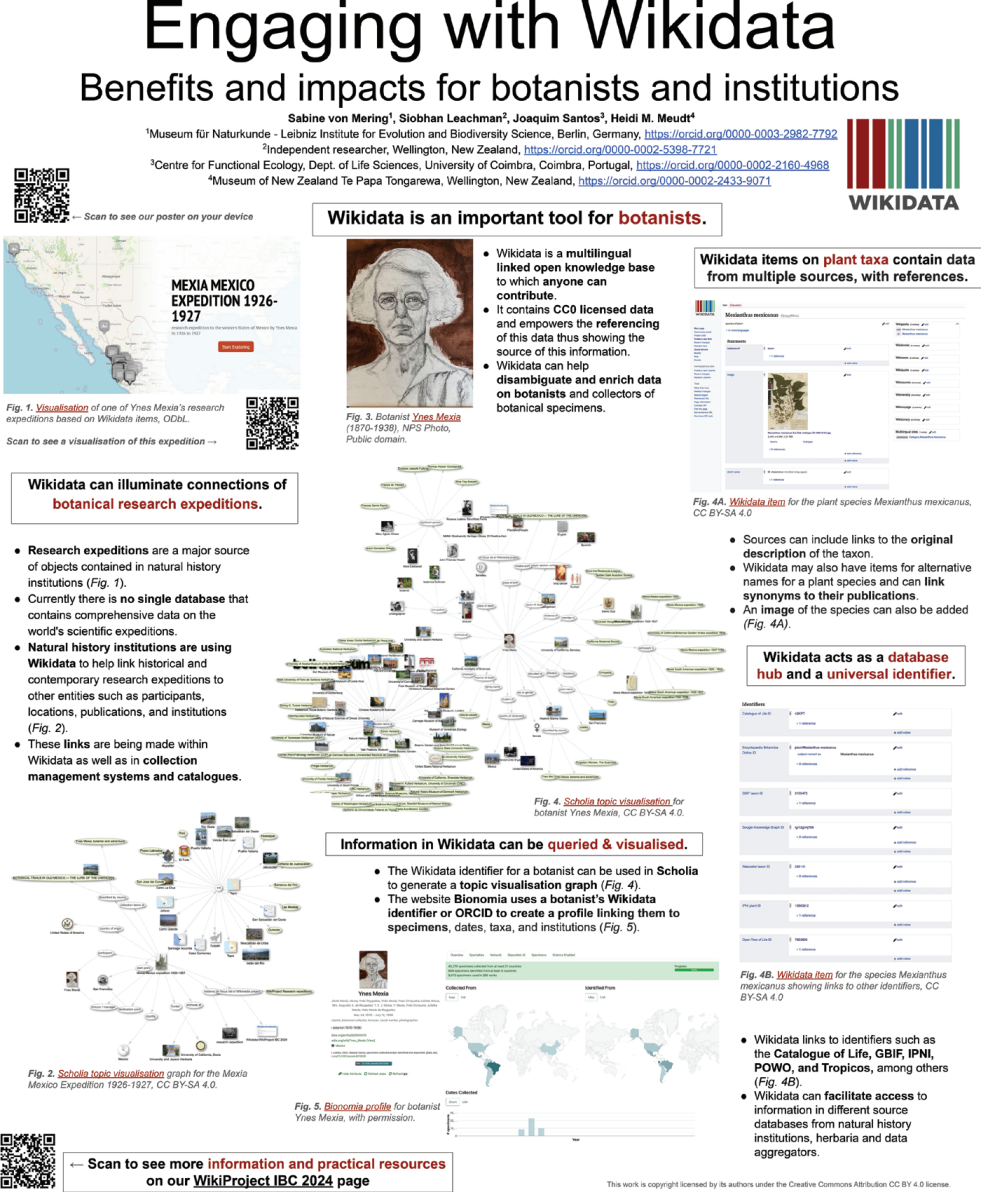
The poster ‘Engaging with Wikidata: Benefits and impacts for botanists and institutions’ (von Mering *et al.*, 2024*a*; https://doi.org/10.5281/zenodo.11102530) presented at the XX International Botanical Congress in Madrid, Spain in July 2024, which led to the collaborative writing and eventual publication of this article. Available online at https://commons.wikimedia.org/wiki/File:IBC2024_Wikidata_poster.jpg, Sabine von Mering, Siobhan Leachman, Joaquim Santos and Heidi Meudt, CC BY 4.0 (https://creativecommons.org/licenses/by/4.0), via Wikimedia Commons.

The current article is the result of an international collaboration of the four co-authors (three of whom are botanists and all of whom are Wikimedians) and their activities at the XX International Botanical Congress (IBC) in Madrid, Spain, in July 2024 (see https://www.wikidata.org/wiki/Wikidata:WikiProject_IBC_2024, date last accessed, 15 May 2025). Our contributions included organizing a full day in-person workshop entitled, ‘Upskilling in Wikidata for maximum impact: A practical workshop for botanists’ (together with online onboarding and follow-up sessions) and a poster entitled, ‘Engaging with Wikidata: Benefits and impacts for botanists and institutions’ (von Mering *et al.*, 2024*a*; Fig. 6), as well as additional presentations and posters on other projects that use Wikidata. All these contributions highlighted the value of Wikidata in botanical research, encouraged botanists to contribute to Wikidata, and led to the collaborative writing and publication of this article.

## GETTING TO KNOW WIKIDATA

**Fig. 7.**
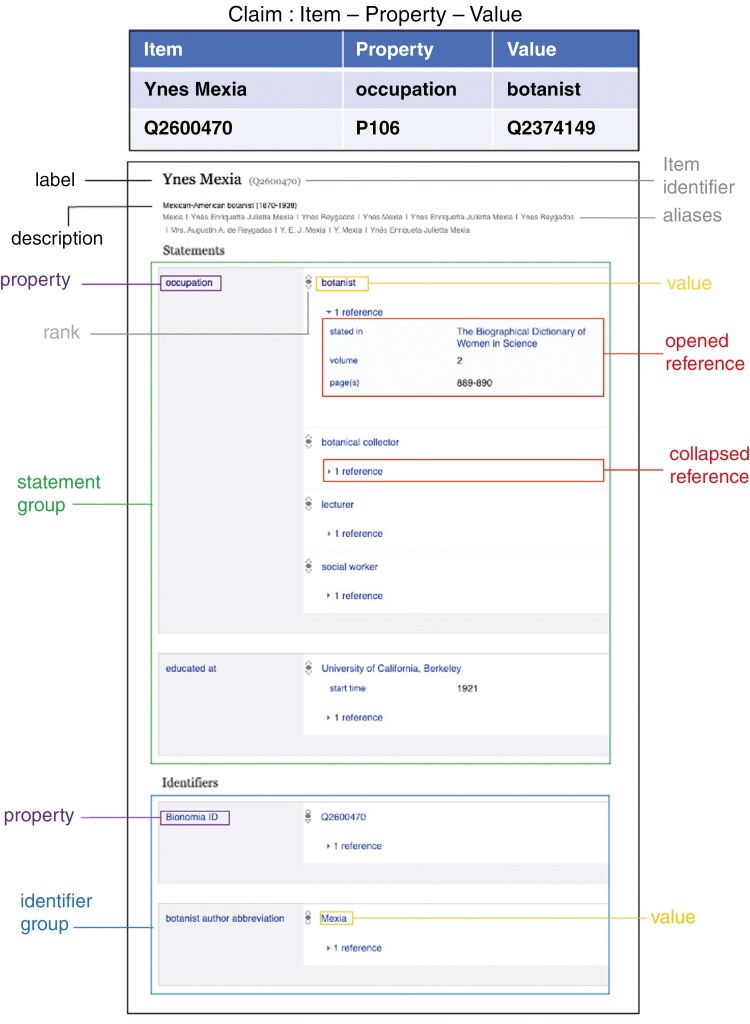
An annotated Wikidata *item* exemplifying different parts of Wikidata, using the Mexican–American botanist Ynes Mexia (1870–1938) as an example. Available online at https://commons.wikimedia.org/wiki/File:Ynes_Mexia_Wikidata_item_example.jpg, Makenzie Mabry, CC BY-SA 4.0 (https://creativecommons.org/licenses/by-sa/4.0), via Wikimedia Commons). For definitions of some Wikidata terms, see https://www.wikidata.org/wiki/Wikidata:Glossary.

Wikidata is a multilingual, linked, free and open knowledge base that is community-curated and maintained; anyone can contribute to it (e.g. Vrandečić and Krötzsch, 2014; Shafee *et al.*, 2023). While its sister project Wikipedia comprises pages describing people, places and many other things (entities) as written prose in essay-style texts, Wikidata is a database that provides structured data on such entities as a list of referenced statements. The ‘pages’ on Wikidata for each entity are called *items*, and each *item* has a unique identifier that starts with the letter Q, the Wikidata Q identifier, also known as Wikidata QID or Q number (Q43649390; Fig. 7), as well as a *label* and a *description* (Fig. 7; see also https://www.wikidata.org/wiki/Wikidata:Glossary for definitions of these and other Wikidata terms).

At the top of each *item*, both the *item label* and *description* can be given in multiple languages. All data on Wikidata is structured according to a data model that uses *triplets* involving the subject (*Wikidata item*  Q16222597) + predicate (*Wikidata property*  Q18616576) + object (*value*). For example, a sentence like ‘Alicia Lourteig is a botanist’. would be represented in Wikidata as an *item* (Alicia Lourteig) that has a *statement* which consists of a *property* (in this case *occupation*) and a *value* (here botanist; see Q454806). Statements can be further specified by so-called qualifiers (Q15720608), e.g. for an institution she worked for (*employer*, P108) to add qualifiers *start*  *time* (P580) and *end time* (P582) as well as *position held* (P39). Each *item* will have multiple *statements* (Fig. 7). Wikidata allows and encourages the referencing of the source of the data, and *references* to publications, websites or other Wikidata *items* are shown together with *statements* contained in the database (Fig. 7).

As well as Q identifiers for Wikidata *items* and P identifiers for Wikidata properties, Wikidata also has two other identifiers, L for lexemes and E for entity schemas (Shafee *et al.*, 2023; Morshed, 2024). Lexemes precisely define words, their inflections, meanings and other such metadata (Nielsen, 2019). By adding lexemes to Wikidata, structured metadata about words and their meaning in various languages become reusable in tools and queries (Shafee *et al.*, 2023). Lexemes are relevant to botany as they empower distinct scientific terms to be adequately defined in multiple languages. Entity schemas provide models for how data should be added or enriched in Wikidata for various entities. They help ensure data are added in a consistent manner and assist with facilitating community agreement on appropriate data models to be used (Shafee *et al.*, 2023; Morshed, 2024).

Wikidata is multilingual by design and can be read and edited by both machines and humans; in fact, anyone can contribute to Wikidata and make use of the data that are stored there. The data are openly licensed under a Creative Commons Zero 1.0 Universal licence (CC0, https://creativecommons.org/publicdomain/zero/1.0/, date last accessed, 15 May 2025) and thus can be reused without restriction. Importantly, the Wikidata QID acts as an identifier hub linking to other institutions’ databases and information from other sources and platforms via their identifiers (Groom *et al*., 2020). In this way, the Wikidata QID has become a universal identifier. Because of this, there is great potential for ‘roundtripping’ data with other international biodiversity and botanical databases (e.g. BHL, 2025; GBIF, 2025; IPNI, 2025; Tropicos, 2025; WFO, 2025).

Wikidata contains botany-related data such as data on botanists and botanical collectors, botanical taxa, natural history institutions and collections, botany-related publications, geographical locations and research expeditions. Wikidata can also contain other data related to botany, such as genes, genetic variants, chemical compounds, natural products and diseases (Waagmeester *et al.*, 2020). And because these data are linked, Wikidata can be an extremely powerful tool for querying and visualizing both the data and the linkages between them. For example, a Wikidata query can show us on a map how many botanists (or herbaria, Fig. 4; or species) there are in the world (or a particular country), provide lists of research papers about a particular topic, or allow us to quickly find all the available identifiers associated with a particular taxon or person.

Wikidata *items* and their associated data can also be used by third-party tools or websites such as Bionomia (Shorthouse, 2020*a*, *b*) to visualize the collections of a particular (deceased) botanical collector, linking them to institutions, localities, co-collectors, plant taxa, research and papers (for living collectors, instead of Wikidata QIDs Bionomia uses ORCID, Open Researcher and Contributor Identifier; ORCID, 2024). Botanical research expeditions can be visualized (using Expeditia; Santos, 2024), as can botanist collaborator networks (using Scholia; Neilsen *et al.*, 2017).

Of course, the results of such queries depend entirely on the completeness of the information in Wikidata. Here, we use a broad definition of botany to include the study of many different taxa (vascular plants, non-vascular plants, algae, fungi and lichens) and specializations (e.g. taxonomy, genetics, morphology, physiology, ecology, biotechnology, ethnobotany, phytochemistry, etc.). At the moment, most of the effort and botany-related data in Wikidata relates to taxonomy and collections-based data, but there is great potential to enhance Wikidata in other aspects of botany now and in future. There are many ways that we botanists can work to improve the quantity and quality of information related to botany, botanists and botanical collections in Wikidata. The main aim of this article is to provide the background, practicalities and justification for doing so, and a call to action to each of us, and to the botanical community as a whole, to collectively make this happen.

## BOTANY-RELATED DATA IN WIKIDATA

Wikidata comprises *items* for a variety of different entities. This section focuses on information about such entities related to botany as people, taxa, publications, institutions, locations and research expeditions.

### People

The Wikidata QID has been recommended by Biodiversity Information Standards (TDWG, 2025) as an appropriate identifier to use when disambiguating botanists and botanical collectors. The basis of this recommendation was that Wikidata provides a unique identifier for botanists and acts as a database hub assisting with the linking of identifiers generated by disparate organizations (Groom *et al.*, 2022).

**Fig. 8.**
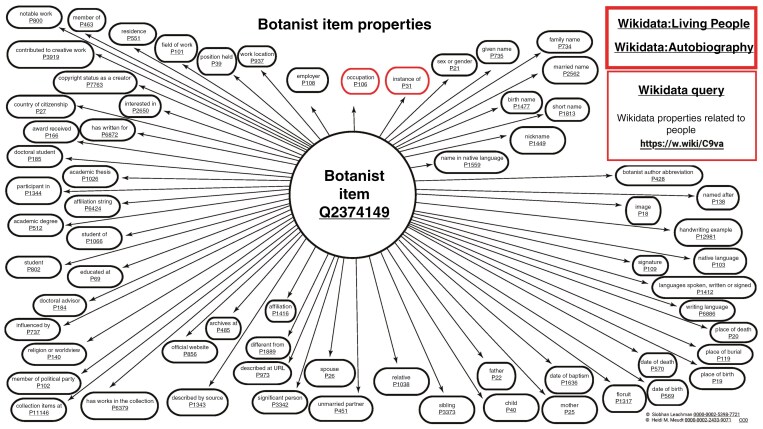
Visualization of a Wikidata data model for a botanist, including over 60 examples of Wikidata *properties* (not including *identifiers*) that can be used on botanist *items* in Wikidata. Available online at https://commons.wikimedia.org/wiki/File:Botanist_item_properties_diagram_10_Dec.jpg, Siobhan Leachman and Heidi Meudt, CC0 (https://creativecommons.org/publicdomain/zero/1.0/) via Wikimedia Commons. A hyperlinked pdf version of the same figure can be found here: https://commons.wikimedia.org/wiki/File:Botanist_item_properties_diagram_10_Dec.pdf.

Wikidata *items* relating to both deceased and living botanists and botanical collectors can contain biographical data, such as their *birth name* (P1477), *name in native language* (P1559), *date of birth* (P569), *date of death* (P570), *employer* (P108) and institutions they were *educated at* (P69) (Fig. 8). Wikidata also collates multiple institutional identifiers relating to people on their *items* such as *BHL creator ID* (P4081), *Bionomia ID* (P6944), *Harvard Index of Botanists ID* (P6264), *IPNI author ID* (P586), *ORCID iD* (P496), *Tropicos person ID* (P12807) and *VIAF cluster ID* (P214), among others. In doing so, Wikidata not only links the *item* to the relevant external institutions, it also provides a bridge or ‘brokerage system’ between different institutions’ knowledge about that person (Groom *et al.*, 2020). This can include institutions that hold the archives, collections or works of that botanist, botanical collector or botanical illustrator.

By undertaking this linking, Wikidata assists with the disambiguation of botanists and botanical collectors, and facilitates the collation and enrichment of data known about these people. This is especially critical for under-represented botanists (Lemus-Rojas and Lee, 2020; Leachman and Mabry, 2024), including women, LGBTQ+, Black, Indigenous and People of Colour (BIPOC) and other minorities, as well as local or indigenous collectors, guides and interpreters, particularly those from the Global South (Auge *et al*., 2024). Building up the Wikidata *items* for under-represented botanists can be a first step towards aggregating verifiable, reliable, independent sources to achieve the notability criteria for creating Wikipedia articles about them (Martini, 2023). In addition, knowing who we are as a botanical community will allow us to connect, collaborate and utilize our human diversity.

Wikidata can link people to data such as their occupations, field of work, educational information, employment history, work locations, scientific academy or society memberships and roles, archival and collection repositories, and publications (von Mering *et al.*, 2024*b*). Different professions, positions and specializations can be added to the Wikidata property *occupation* (P106). A typical Wikidata *item* of someone active in botany could include *occupation* as botanist (Q2374149), botanical collector (Q2083925) or curator (Q674426) among others, while some of these could also be added instead as a qualifier, e.g. *position held* listed as curator or editor (Q1607826) for a certain employer. For historical botanists additional information may also be included under *occupation*, including explorer, naturalist or (in colonial contexts) colonial administrator or military personnel (von Mering *et al.*, 2024*b*). In addition, the property *significant person* (P3342) can be used to connect to relevant persons and add the qualifier *object of statement has role* (P3831) specifying their relationship, e.g. co-author (Q15735983), co-collector (Q81546212), colleague (Q18029574), correspondent (Q3589290) or friend (Q17297777) (von Mering *et al.*, 2024*b*). These data can then be used to create a relationship network for the botanist or botanical collector. Other relevant properties include *collection items at* (P11146), *archives at* (P485) and *has works in collection* (P6379), which are used to specify where specimens collected by a botanist, archival materials, or works of art (including botanical illustrations) are held. Over 60 examples of Wikidata *properties* (not including *identifiers*) that can be used on botanist *items* are shown in Fig. 8.

### Taxa

**Fig. 9.**
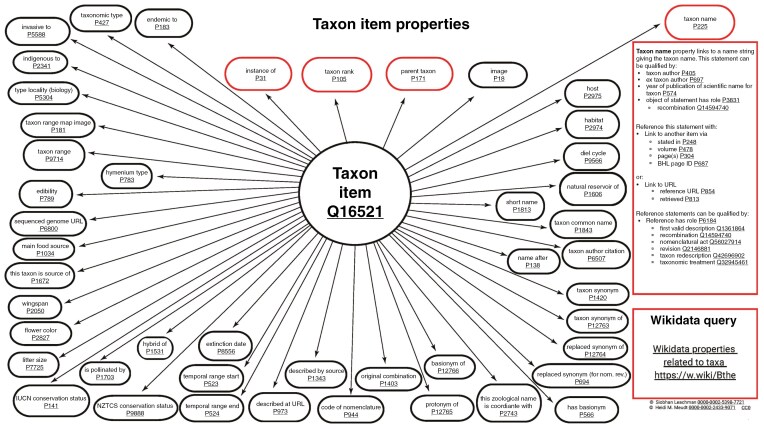
Visualization of a Wikidata data model for a taxon, including nearly 50 examples of Wikidata *properties* (not including *identifiers*) that can be used on taxon *items* in Wikidata. Available online at https://commons.wikimedia.org/wiki/File:Taxon_item_properties_diagram.jpg, Siobhan Leachman and Heidi Meudt, CC0 (https://creativecommons.org/publicdomain/zero/1.0/), via Wikimedia Commons. A hyperlinked pdf version of the same figure can be found here: https://commons.wikimedia.org/wiki/File:Taxon_item_properties_diagram_pdf.pdf.

Wikidata *items* relating to a taxon (Q16521) can contain taxonomic information about a taxon at any rank. A typical Wikidata *item* of a taxon could include *taxon name* (P255), *taxon rank* (P105), *parent taxon* (P171), *taxon author citation* (P6507), *described by source* (P1343), *taxon synonym* (P1420), *taxon common name* (P1843; in multiple languages), *taxon range* (P9714), *sequenced genome URL* (P6800), *image* (P18), and many others, all of which can be referenced to multiple sources, including publications, databases and other links. Nearly 50 examples of Wikidata *properties* (not including *identifiers*) that can be used on taxon *items* are shown in Fig. 9.

Taxa are described using names as a proxy for a concept. Because taxonomy and nomenclature are dynamic, names and concepts can change over time: the same concept can have multiple names and vice versa. It has been debated how to address this through the use of identifiers for taxon concepts, rather than taxon names (Geoffroy and Berendsohn, 2003; Remsen, 2016). Despite some successful implementations in other systems (Lepage *et al.*, 2014; Hüllbusch *et al.*, 2024), this approach has not been adopted by Wikidata. Nevertheless, the use of properties such as *taxon synonym* (P1420) and its inverse property *taxon synonym of* (P12763), as well as the pair *replaced synonym of* (P12764) and *replaced synonym (for nom. nov.*) (P694) can assist in tracking the mutability of taxon names.

Wikidata also collates and links to multiple identifiers relating to taxa on their *items*, including *Catalogue of Life ID* (P10585), *GBIF taxon ID* (P846), *iNaturalist taxon ID* (P3151), *Invasive Species Compendium Datasheet ID* (P5698), *IPNI plant ID* (P961), *NCBI taxonomy ID* (P685), *Open Tree of Life ID* (P9157), *Plants of the World Online ID* (P5037), *Tropicos ID* (P960), *USDA PLANTS ID* (P1772), *World Flora Online ID* (P7715), *WoRMS-ID for taxa* (P850) and dozens of others (see e.g. potato, Q10998) (Shafee *et al.*, 2023). Through these links, Wikidata assists with the understanding and visualization of a taxon name and the enrichment of data known about that taxon. Wikidata can aid the enrichment of taxon-related articles in multiple language Wikipedias. For example, the identifiers from the Wikidata *item* for a species (such as *Oxalis psoraleoides*, Q131350563) are pulled into its Wikipedia article (https://en.wikipedia.org/wiki/Oxalis_psoraleoides, date last accessed, 15 May 2025) using a template that creates a ‘Taxon Identifiers’ box at the bottom of the page.

As with people described above, digital tools can also use the data contained in Wikidata to enrich websites, databases and catalogues about these taxa. For example, the Atlas of Living Australia (ALA) ingests both the taxon’s Wikipedia article and its accompanying Wikidata-generated taxon identifier data into the appropriate taxon page for a species, genus, family, etc. (Belbin *et al.*, 2021). The *External links* section on the bottom left of the ALA page for Oxalidaceae R.Br. is a good example (Atlas of Living Australia, 2024).

Wikidata can compile and reflect multiple taxonomies and classifications of a taxon, as multiple and differing referenced *statements* can be made for various *properties* on the taxon *item*. Wikidata also has separate *items* for alternative names for a taxon and can link synonyms to associated taxonomic names and their supporting publications and databases (Fig. 8). In this way, Wikidata helps to correct ‘a major gap in the biodiversity knowledge graph’ which is ‘a connection between taxonomic names and the taxonomic literature’ (Page, 2023).

Wikidata can also be used as a research tool to query, understand, improve and research a particular topic in botany. For example, an innocent question on social media (‘How many plant genera were actually named after women?’) led to a multinational collaboration that answered this question by creating a multi-linked dynamic dataset in Wikidata (von Mering *et al.*, 2023*a*). The authors linked the Wikidata *item* for an eponymous plant genus with that of the woman (or mythical female being) after whom it was named using referenced statements in the property *named after* (P138), thus linking between people, publications and taxa for eponymy to find the answer: at least 728 angiosperm genera were named after women or female beings (von Mering *et al.*, 2023*a*).

Finally, although we do not advocate the creation of Wikidata *items* for all specimens, it is possible for type specimens to be added to link identifiers related to types. As at the writing of this paper, this has not been worked on extensively in the Wikidata community.

### Publications

Wikidata is also a massive database of scholarly publications, with nearly 45 million *items* that are categorized as scholarly articles (see Wikidata query https://w.wiki/ESSb, date last accessed 11 June 2025). Scholarly article (Q13442814) is by far the most common *item* in Wikidata, constituting ~40 % of all Wikidata *items* (Fig. 3).

A huge opportunity exists to link publications to authors, their affiliations, taxa, main subjects and other publications (such as those cited inside the scholarly article). Over one million taxonomic names (of all organisms) are linked to at least one Wikidata *item* for a publication, making Wikidata the largest source of taxa linked to a publication with a persistent *identifier*, i.e. a Wikidata QID (Page, 2023).

The linking of publications to all these other *items* can greatly improve scientific efficiency. For example, linking a taxon Wikidata *item* to the Wikidata *item* for the publication that contains the original description of the species allows the digitized version of the taxon description to be only one click away. To add scientific publications to Wikidata, one simply pastes the publications’ DOIs (digital object identifiers) into the search field of a very useful tool created by Roderic Page (https://bhl2wiki.herokuapp.com/, date last accessed, 15 May 2025). The tool checks to see if those DOIs already exist in Wikidata. If no *item* exists, the tool will automatically create a set of text commands for QuickStatements (another tool for batch-editing Wikidata; Q20084080; https://quickstatements.toolforge.org/#/, date last accessed, 15 May 2025) to enable anyone to add the publication to Wikidata as well as links to its cited references (if those publications already have *items* in Wikidata). This tool will add the following *statements* to the Wikidata *item* of a publication: *title* (P1476), *author* (P50) or *author name string* (P2093), *publication date* (P577), *work available at URL* (P953), *published in* (P1433) and *cites work* (P2860). Manually adding additional information to the publication Wikidata *item*, including statements such as *main subject* (P921; e.g. keywords, taxa, field of work) and *type of publication* (P13046; e.g. research article, review article, monograph, citizen science, data paper, obituary, type catalogue) can help botanists to query, visualize and utilize these additional linkages to other related papers and authors in their own research. Linking the Wikidata *item* for a publication to the Wikidata *items* for all its cited references makes the finding of these relevant resources much easier and faster, and improves attribution, visibility and linkages of the botanists authoring these papers (Lemus-Rojas and Lee, 2020; Lemus-Rojas *et al.*, 2022; Leachman and Mabry, 2024).

### Institutions

Wikidata *items* already exist for many organizations and research institutions (*research institute*, Q31855; https://www.wikidata.org/wiki/Special:WhatLinksHere/Q31855, date last accessed, 15 May 2025) in the field of botany, including universities, natural history museums, herbaria, botanical gardens, DNA banks, seed banks, laboratories and biological research stations. However, while Wikidata *items* often exist for larger parent organizations, *items* are often still needed for the collections that are part of them (e.g. herbaria that are part of a larger institution, such as a university, botanical garden or museum). These *items* can provide information on the institutions—such as year of *inception* (P571), *employees* (P1128), *coordinate location* (P625), *official website* (P856) and other links to Wikidata *items* giving historic names of the institution—as well as other information, such as visitors per year or number of followers on social media channels.

Wikidata *items* for institutions can also link to different external identifiers, such as the *Index Herbariorum code* (P5858), the *Biodiversity Repository ID* (P4090) linking to GBIF’s GRSciColl (2024), and the *ROR ID* (P6782) of the Research Organization Registry (ROR, 2025). Wikidata’s function as a hub for identifiers is very important as it facilitates transparency and accessibility of natural history collections, including herbaria and links to institutional metadata and datasets in different source databases and data aggregators. Wikidata has the potential to improve the visibility of institutions and their collection data, and make their collections, archival material and research output more discoverable (e.g. von Mering *et al.*, 2024*b*). However, very few herbaria are currently in Wikidata, and of these even fewer have coordinates to allow mapping (see Wikidata query https://w.wiki/CU4r; Fig. 4).

To date there has been no single data model that has been strictly followed for the creation and linking of Wikidata *items* for institutions to their constituent parts. The authors encourage the Wikidata community to agree on a single data model for creating and linking of institution *items*, e.g. by engaging with appropriate Wikidata WikiProjects, where this topic can be discussed and resolved.

### Locations

Wikidata *items* for *geographic locations* (Q2221906) are useful for many purposes related to botanical research. The most obvious is to link to places, countries or regions where a botanist was active, such as *work location* (P937), and to the *place of birth* (P19) and (for deceased botanists) *place of death* (P20). *Items* for locations can link, for example, to historic and current place names using properties such as *official name* (P1448) and *native label* (P1705), *coordinate location* (P625), *image* (P18), related administrative units, e.g. *located in the administrative territorial entity* (P131), and identifiers in databases such as *GeoNames ID* (P1566), *iNaturalist place ID* (P7471) and *OpenStreetMap node ID* (P11693). Wikidata can also support georeferencing and linking of type localities, the places from which new taxa were first described (based on type specimens). It can also provide and connect information on a particular *biological station* (Q113288787) or *research station* (Q195339). For historical contextualization, e.g. of collecting sites from colonial times, Wikidata already provides some information on such locations as well as *mission stations* (Q1564373) and various types of military outposts.

### Research expeditions

Research expeditions (Q366301) are a major source of objects contained in natural history collections. Such collection events are historically interesting and continue to be relevant today (von Mering *et al.*, 2023*b*, 2024*c*). However, there is currently no single database that both contains comprehensive data on the world’s research expeditions and generates a unique identifier for these expeditions. Wikidata can be used as both a data repository for research expeditions and as a tool to provide identifiers for these research expeditions. The Wikidata QID for such research expeditions (as is the case for people, discussed above) can be used by natural history institutions in collection management systems and catalogues (Leachman and Schrader, 2024).

**Fig. 10.**
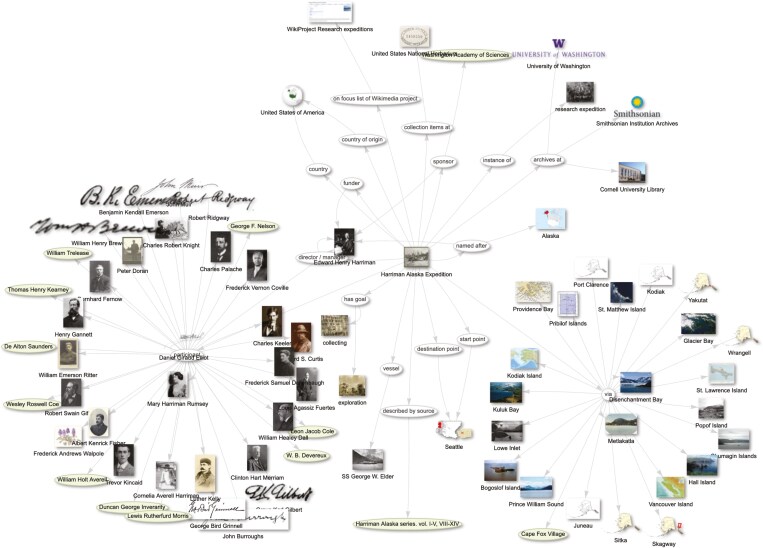
Knowledge graph of Harriman Alaska Expedition created via Scholia (https://scholia.toolforge.org/topic/Q1586167#context). Available online at https://commons.wikimedia.org/wiki/File:Scholia_Knowledge_graph_of_Harriman_Expedition.jpg, Wikimedia Foundation, CC BY-SA 4.0 (https://creativecommons.org/licenses/by-sa/4.0), via Wikimedia Commons.

**Fig. 11.**
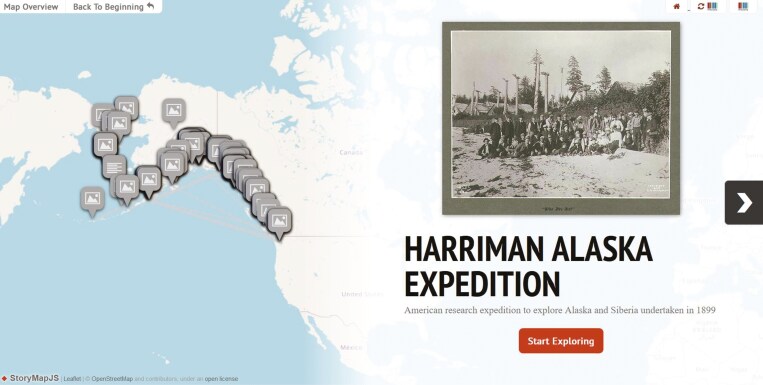
Screenshot from the tool Expeditia showing the itinerary of the Harriman Alaska Expedition (https://expeditia.info/?QID=Q1586167). Available online at https://commons.wikimedia.org/wiki/File:Expeditia_visualisation_of_Harriman_Alaska_Expedition.jpg, Joaquim Santos, Expeditia.info, ODbL (http://opendatacommons.org/licenses/odbl/1.0/), via Wikimedia Commons.

Wikidata can assist in illuminating connections between research expeditions and many other entities using properties such as *country of origin* (P495), *country* (P17), *participant* (P710), *vessel* (P1876), *collection items at* (P11146), *archives at* (P485) and *described by source* (P1343) (Fig. 10). Furthermore, Wikidata can be used to create itineraries of research expeditions using properties like *start point* (P1427), *via* (P2825) and *destination point* (P1444) that can then be visualized (Fig. 11).

Adding research expeditions to Wikidata facilitates the linking of the specimens collected during these expeditions, which today may be widely distributed at multiple institutions throughout the world. The Wikidata *item* for a research expedition becomes a repository of referenced information about a particular research expedition, which can be a way to prepare for (and ascertain notability for) writing a Wikipedia article or scholarly work about it. Currently, the TDWG Task Group Modelling Research Expeditions (https://www.tdwg.org/community/cd/expeditions/, date last accessed, 15 May 2025) is working together with Wikimedians involved with the Wikidata WikiProject Research Expeditions (https://www.wikidata.org/wiki/Wikidata:WikiProject_Research_expeditions, date last accessed, 15 May 2025) to create a research expedition schema, produce best practice documentation, and add relevant research expedition data to Wikidata (von Mering *et al*., 2023*b*, 2024*c*).

## REUSE OF DATA FROM WIKIDATA

Since all data in Wikidata are shared under the open CC0 licence, Wikidata QIDs and their associated data can be reused by anyone for any purpose. Below, five examples of digital tools in the Wikiverse are highlighted that use data from Wikidata to create data visualizations. Then, three third-party institutions that make use of Wikidata QIDs or their associated data in tools, websites or catalogues are briefly discussed. All of these will be of interest and relevant to botany, botanists and botanical institutions.

### Scholia

**Fig. 12.**
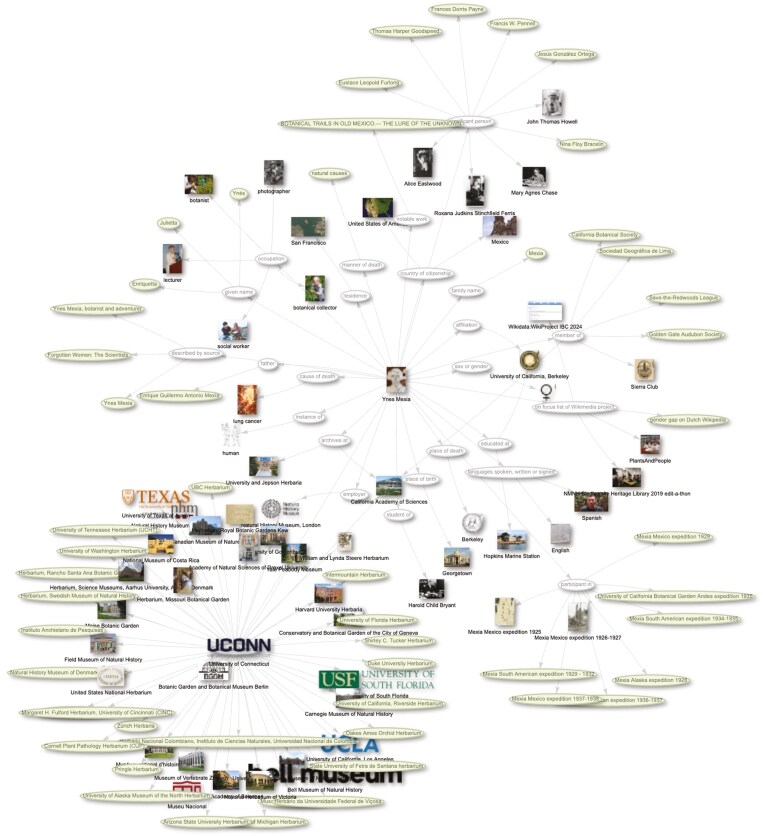
Knowledge graph of Ynes Mexia created via Scholia (https://scholia.toolforge.org/topic/Q2600470#context) showing the research expeditions she has participated in, her significant person network, the institutions that hold her collections and other connected data. Available online at https://commons.wikimedia.org/wiki/File:Ynes_Mexia_Scholia_topic_in_context_visualisation.jpg, Wikimedia Foundation, CC BY-SA 4.0 (https://creativecommons.org/licenses/by-sa/4.0), via Wikimedia Commons.

Scholia is a powerful Wikidata visualization tool that can also work in conjunction with other tools to edit Wikidata (Neilsen *et al.*, 2017; Lemus-Rojas *et al.*, 2022). Scholia is just one example of a tool developed by the Wiki community to build on and reuse data from Wikidata. As an example of its use, when a botanist has a Wikidata *item* (e.g. Ynes Mexia Q2600470), Scholia can use the linked data from this *item* to visualize these data and to facilitate the linking of that botanist to their publications, collections, associated people, institutions, research expeditions, and so on (Fig. 12). In this way, Scholia creates scholarly profiles that reveal the multiple and diverse impacts of a botanist during their career. Scholia has a similar function to other online platforms and products but can be distinguished from them in its commitment to openness, i.e. it is based on free and open software and data that can be accessed on any web browser and used for any purpose (Rasberry *et al.*, 2019; Lemus-Rojas *et al.*, 2022).

### TL-2

The digital version of Taxonomic Literature, edition 2 (TL-2) has linked all taxonomic authors listed in that publication to their Wikidata *items* (tl2.io, 2025). This work can be extended in Wikidata with publications being linked to their authors and the taxa named within them. Botanists can join the Wiki community to ensure all TL-2 data are available as Linked Open Data in Wikidata, and can enrich it further by adding updated and novel information concerning these subjects, as TL-2 stopped publication in 1940. Doing so ensures the most updated TL-2 data are openly available and easily accessible to any botanist doing taxonomic research.

### Bionomia

The website and tool Bionomia (Shorthouse, 2020*a*, *b*) links natural history specimens published in GBIF to the world’s collectors and to those people who have identified that material. Bionomia uses Wikidata QIDs (for deceased collectors) or ORCID IDs (for living collectors) to create profiles linking botanists, natural history collectors and other scientists to specimens they have collected or identified. Where institutions already record and upload these identifiers to GBIF via their collection management systems using Darwin Core terms dwc:recordedByID and dwc:identifiedByID (Darwin Core, Q5225953), these links are made automatically. When the identifiers are not provided, volunteers (called scribes) help attribute specimens to people manually or by using tools integrated in Bionomia; anyone with an ORCID ID can act as a scribe in Bionomia.

Bionomia provides visualizations of collections of natural history collectors, linking them to institutions, countries, co-collectors, taxa, research and papers (Shorthouse, 2020*a*, *b*). Wikidata identifiers are integrated in Bionomia to capture the names, including other variants and abbreviations and further information. Properties used to gather data include *name in native language* (P1559) and the aliases (from the *also known as* field tagged as having languages English ‘en’ and the default for all languages ‘mul’), *employer* (P108), *affiliation* (P1416) as well as *date of birth* (P569) and *date of death* (P570) for deceased botanists or botanical collectors. Thus, adding more aliases to the Wikidata *item* facilitates the ease of attributing specimens in Bionomia.

As a result of this linking, Bionomia is used to ‘maximize downstream data integration, engagement, and as a means to discover errors or inconsistencies in natural history specimen data’ (Shorthouse, 2020*a*). In addition, it can provide packages of Frictionless Data (https://frictionlessdata.io/, date last accessed, 15 May 2025) for download. Collection managers can use these zipped, UTF-8-encoded relational files to help to improve the collection data of their institution (Shorthouse, 2020*a*; see https://en.bionomia.net/collection-data-managers, date last accessed, 15 May 2025).

Botanists and institutions can cross-validate information from Bionomia (such as imported specimen data from GBIF) with Wikidata statements to improve the quality of shared collection data. For example, if there are inconsistencies between certain statements in the Wikidata *item* for a collector and their linked Bionomia profile, this may indicate an issue with the disambiguation of the person or with the specimen data, which can then be corrected or updated in collection management systems, Wikidata and other databases. Bionomia profiles show that a person did (or—similarly important—did not) work in a certain region or country and can thus support plausibility checks.

### Expeditia

Botanical research expeditions can be visualized using Expeditia, a tool that displays research expedition itineraries and the natural history specimens gathered during these events (Santos, 2025). It queries Wikidata periodically to get a list of research expeditions. For each of these, the tool retrieves the *start time* (P580), *start point* (P1427), *end time* (P582), *destination point* (P1444) and *via* (P2825) for a list of locations visited during the expedition. Expeditia then queries GBIF to obtain specimens that use the Wikidata QID of the expedition as an identifier for the Darwin Core terms dwc:eventID or dwc:parentEventID. The tool also uses the people listed on the expedition *item* via the property *participant* (P710) to query Bionomia for the specimens collected by them on dates coinciding with the expedition dates. Finally, Expeditia then plots on a map all the places and specimens that have coordinates showing the places visited and the specimens collected (Fig. 11).

### LOTUS

The LOTUS initiative is a database of natural product occurrences hosted by Natural Products Online, an open source and open data portal for natural products cheminformatics. LOTUS makes use of Wikidata for chemical entities, organisms’ taxonomy and scholarly articles, thus allowing community curation and enrichment of data. In addition to other sources, it aggregates data from Wikidata, displayed in a user-friendly interface referencing structure–organism pairs and establishing relationships between molecular structures and the organisms from which they were identified. It allows simple and advanced searches, exports, and also provides an Application Programming Interface (API), allowing machine interoperability (Rutz *et al.*, 2022*a*, *b*).

### Biodiversity Heritage Library

BHL has added links to appropriate Wikidata *items* onto the BHL creator pages in their catalogue. BHL has used these Wikidata *items* to extract other third-party identifiers to add to and enrich the BHL catalogue (Dearborn and Leachman, 2023). This integration into the BHL catalogue assists the botanical community to more easily find relevant information about the creators of that content.

### CETAF Botany Pilot

In recent years, institutions of the Consortium of European Taxonomic Facilities (CETAF, Q5163385) have started to semantically annotate and enrich their collection data with links to related resources to enable the unifying of distributed collections. The CETAF Botany Pilot (Q131471435), a collaboration of five large European herbaria, is an example of this approach to make diverse data relating to collectors of botanical specimens available by linking to stable people identifiers. Wikidata QIDs are the identifiers used for people in this pilot project (Güntsch *et al.*, 2021). Using this single-person identifier allows institutions to connect and dynamically pull together botany-related data, including biographical information from Wikidata, specimens in different herbarium collections, publications in BHL, and more recently also entries from Bionomia (Güntsch *et al.*, 2021). As a result, data on collectors can be queried across different collections, thus making the material collected by them more accessible, including for researchers and other stakeholders in the Global South.

### Te Papa Collections Online

The Museum of New Zealand Te Papa Tongarewa (Te Papa, Q915603) is one example of an institution that has committed to linking Wikidata *items* for entities such as people and research expeditions into their collection management system. This facilitates the linking to the appropriate Wikidata QID being shown on the Te Papa Collections Online website (Leachman and Schrader, 2024). It also facilitates the disambiguation of botanical collectors, which are then automatically matched via their Wikidata QID when the museum’s specimen data are uploaded to GBIF and imported to Bionomia. By using the Wikidata QID as a unique stable identifier for both botanists as well as research expeditions, Te Papa can increase the recognition given to these botanists and the research expeditions they participate in for contributions made.

## BENEFITS OF WIKIDATA FOR THE BOTANICAL COMMUNITY

Wikidata has multiple benefits for the botanical community. It already comprises a wealth of valuable botany-related information. Using this existing information, and enriching and further linking it, has practical advantages for botanists and botanical institutions.

Wikidata can assist in increasing the visibility of botany, botanists and plants and can help by filling gaps in knowledge relating to each of these subjects. Wikidata also has the potential to improve the visibility of early-career researchers and established botanists by linking them to their research, publications and collaborations in an easily accessible and reusable format in an open environment, therefore making their contributions more accessible and usable (Lemus-Rojas and Lee, 2020; Lemus-Rojas *et al.*, 2022; Leachman and Mabry, 2024). Wikidata can extend the reach of a particular botanist’s publications and their network, especially via Scholia (see Reuse of data from Wikidata, above). This can be significant for those less visible on other platforms or underserved by them (Lemus-Rojas *et al.*, 2022). Many organizations use data from Wikidata because they can be integrated into other open infrastructure services, platforms and technologies, including Google and artificial intelligence (AI) (Lemus-Rojas *et al.*, 2022). Thus, including more botanists in Wikidata ensures their inclusion and accurate representation in downstream applications and increases the collective visibility of their respective institutions (Lemus-Rojas *et al.*, 2022) and the botany field as a whole. Wikidata is a tool that can assist in making marginalized botanical agents more visible and highlight their contributions, e.g. researchers from less well represented regions such as the Global South, as well as those who are women or from the LGBTQ+ community. For example, the Science Stories Collective creates from Wikidata—via a web application—visual profiles and narratives of female scientists, which highlight their contributions in scientific fields that are often male-dominated (Thornton and Seals-Nutt, 2018; Thornton *et al.*, 2022; Centelles and Foran, 2024).

Wikidata also increases the accessibility of knowledge about botany, botanists and plants. Wikidata is part of the Semantic Web (Berners-Lee *et al.*, 2001; Erxleben *et al.*, 2014), with the data contained in Wikidata being part of a wider knowledge ecosystem that is dynamic and continues to expand and be enriched over time by the community. By linking and enriching botanical knowledge into the wider knowledge graph, Wikidata improves the accessibility of that botanical knowledge. This accessibility is also facilitated by the multilingual nature of Wikidata, which retains and increases access to knowledge from multiple languages (e.g. botanical publications, common names of plants).

Wikidata is a valuable database that botanists can query to find information but also to identify data gaps. Wikidata provides data in a human- and machine-readable form, which can be queried manually and using the Wikidata Query Service (Q20950365). As shown above, many tools reuse data from Wikidata and build on their data to visualize or compile data (e.g. Nielsen *et al.*, 2017; Shorthouse, 2020*b*).

Wikidata increases the connectedness of institutions, botanists and botanical knowledge. By empowering institutions and other such identifiers to be linked on *items,* Wikidata increases the interoperability of botanical data. It acts as an identifier hub, bridging between one source of botanical knowledge and other sources of botanical knowledge (Groom *et al.*, 2020). This is why Wikidata is not a *replacement* for other identifiers or platforms (such as ORCID or social media accounts), but rather an important connector which amplifies visibility via a robust online presence (Leachman and Mabry, 2024). Wikidata also increases the discoverability of a researcher’s identifiers and accounts, and engagement with their works (Odell *et al.*, 2022).

Wikidata also assists in the discovery of historical and current connections by linking the past with the present via Wikidata *items* for concepts over time. Furthermore, Wikidata improves the connectedness between communities by encouraging collaboration both within the Wiki community and with the institutions or other organizations or individuals providing the data being added to Wikidata (Groom *et al.*, 2020).

Wikidata can also be used to improve the data quality in other databases and networks, such as natural history collection management systems (Groom *et al.*, 2020). By using Wikidata QIDs to link to stable and unique identifiers for specific entities, Wikidata encourages the enrichment and reuse of both the data related to these entities and the unique identifiers associated with them. This is particularly relevant for botanical collections as Wikidata provides QIDs for such entities as botanical collectors, other collection agents, research expeditions, and so on. A large ecosystem of digital tools to facilitate the enrichment of data has been created by the Wikimedia community and can be utilized by the botanical community to assist in enriching and extracting botany-related data. Examples of these tools include Mix’n’match (Q28054658), QuickStatements, Scholia, Bionomia and the Wikidata Query Service. Another important tool is OpenRefine (Q5583871), which is used for cleaning, reforming and extending data, and, because it also has a Wikidata reconciliation service, can be used by the botanical community to import and enrich data in Wikidata.

Wikidata can be used by the botanical community for quality control purposes, which enables cross-validation and the checking of inconsistencies in data, as well as ensuring the data are plausible. Digital tools can utilize Wikidata to facilitate this quality control process. An example of one such tool that can be used in conjunction with Wikidata to undertake such work is Bionomia (see above). As just one example of quality control, a Bionomia profile for a botanist can show the plant groups or families that a botanist specializes in. This can be cross-checked against the data in Wikidata linked to via the *field of work* (P101) property. Similarly, it is possible to compare information from specimens attributed in Bionomia to data held in Wikidata about the work location for that botanist.

Making use of Wikidata empowers both individual botanists and botanical institutions to increase the visibility of their contributors, improve the accessibility of the botany-related knowledge contributed, assist research capabilities through querying Wikidata, and serve as a tool to facilitate improvements to botany-related data quality.

## OUTLOOK AND CALL TO ACTION

Wikidata may never be fully complete, but the more botanical institutions and botanists contribute to it (including the people reading this article), the more useful Wikidata will become to everyone in the botanical (and wider) community. Therefore, the co-authors of this paper encourage members of the botany community—both individuals and organizations—to add botany-related data to Wikidata and reuse it.

The following section outlines practical guidelines and resources on how the botanical community can enrich Wikidata, including the importance and impact of collaboration on multiple levels, how to get started, data roundtripping by botanical institutions, and the use of Wikidata in the classroom. Next, several ethical and legal considerations that must be considered when editing and using Wikidata are outlined and discussed. Finally, in a call to action, the writers encourage botanists and their institutions to enrich and use Wikidata to achieve the ten strategic actions of the IBC Madrid Declaration (Table 1).

**Table 1.** How botanists can use Wikidata to support the ten calls to action in the Madrid Declaration. For each of the ten calls to action in the Madrid Declaration reproduced in the first column, we have created a complementary call to action for specific ways botanists can use Wikidata to help support each strategic goal. The Madrid Declaration was collectively published by the congress participants at the end of the IBC and is aimed at botanists, institutions and citizens to ‘strengthen the connection between plants and people, nurture mutual benefits, and enhance planetary health and resilience’ (XX International Botanical Congress, 2024).

Call to action (Madrid Declaration)	Call to action for botanists using Wikidata
Plant Diversity as the Foundation: participants call for improved support and recognition of the critical role of plant diversity studies, natural history collections, and botanical gardens.	Add all herbaria, collections, research institutions, universities, natural history museums, botanical gardens, DNA banks, seed banks, laboratories and biological research stations into Wikidata.
Botanical Education at all Levels: participants call for increased emphasis on botanical education from early childhood through lifelong learning.	Add all plant taxa and botanical literature into Wikidata and use open educational resources aimed at integrating Wikidata in the classroom.
Collaborative Transdisciplinary Approaches: participants call for more collaborative and transdisciplinary approaches to plant research, including local and indigenous knowledge, the arts, humanities and diverse scientific approaches.	Work together on collaborative and multidisciplinary Wikidata projects to link botanists, plant taxa, publications, institutions, locations, research expeditions and related resources. Such collective action will help others and lead to botanical or other research follow-up projects, such as the current paper, which was based on Wikidata WikiProject IBC 2024 (https://www.wikidata.org/wiki/Wikidata:WikiProject_IBC_2024). Link information from TL-2 and Bionomia to Wikidata properties for collectors and illustrators at natural history institutions.
Addressing Inequalities in the Plant Sciences: participants call for mutually respectful, equitable, partnership-based approaches and proper inclusion and benefit sharing for all stakeholders involved in research, policy formulation and product development.	Focus on creating, improving and linking Wikidata *items* for under-represented botanists—including women, LGBTQ+, BIPOC, indigenous, and other minorities—as well as under-represented institutions and collections, particularly those from the Global South. Follow ethical and legal guidelines for contributing such data to Wikidata.
Recognizing Biocultural Diversity: participants call for improved recognition and support for biocultural diversity, including the co-production of knowledge and bringing together diverse knowledge systems, methodologies and stakeholders.	Harness our botanical, cultural and language knowledge to add multilingual information, translations, links and references to Wikidata *items* for botanists, plant taxa, institutions, publications, locations of research expeditions and resources. Offer and participate in multilingual Wikidata workshops, videos and online resources, particularly in the Global South.
Plant Diversity is Central to Ecosystem Protection and Restoration: participants call for conservation and restoration strategies that prioritize plant diversity while protecting the functioning of ecosystems and landscapes.	Add all national and regional parks, reserves, and other areas in need of protection or restoration to Wikidata, and link to locality coordinates, habitat information, etc., and legal, policy and research publications supporting protection and restoration of these ecosystems.
Policy Based on Sound Knowledge About Plants: participants call for evidence-based decision-making with improved integration of botanical knowledge into sustainable long-term policy decisions.	Use Wikidata to help facilitate multilingual and multidisciplinary collaborations to improve the accessibility and reusability of botanical knowledge including for policy decisions. Where possible, publish papers and reports as Open Access as Wikidata can amplify the impact of these publications to ensure sound policy decisions.
Harnessing Nature-based Solutions: participants call for the increased recognition and implementation of diverse nature-based solutions that maximize species diversity, increase resilience to climate change, enhance plant conservation and encourage sustainable management and ecosystem restoration.	Participate in Wikidata WikiProject Climate Change (https://www.wikidata.org/wiki/Wikidata:WikiProject_Climate_Change) and related WikiProjects to increase recognition and implementation of nature-based solutions.
A Stronger Role of Plants in Achieving Sustainability and a Net Zero Economy: participants call for an increased recognition of the role of plants in achieving sustainability and establishing a net-zero economy.	Use Wikidata to collaborate with other Wikimedians working on the UN Sustainable Development Goals, including joining Wikimedians for Sustainable Development (https://meta.wikimedia.org/wiki/Wikimedians_for_Sustainable_Development).
Increasing Awareness of the Centrality of Plants for Planetary Health and Resilience: participants call for increased awareness of the importance of plants for planetary health and resilience.	Improve the visibility of plants in Wikidata through engagement with appropriate Wikidata WikiProjects (e.g. https://www.wikidata.org/wiki/Wikidata:WikiProject_Botany and https://www.wikidata.org/wiki/Wikidata:WikiProject_Biodiversity). Use Wikidata to promote planetary health information (e.g. Matos *et al*., 2024; the LOTUS initiative, Rutz *et al.*, 2022*a*).

### Collaboration is key—in botany and in Wikidata

At the XX International Botanical Congress, a distinguished plant taxonomist at the Natural History Museum, London, Sandra Knapp (Q276405), gave the opening lecture, entitled ‘Why botany? Why now?’ (Knapp, 2024). Her inspirational, passionate and forward-looking presentation highlighted how fundamental plants are to the earth, and to us. Importantly, she focused her talk on the key role of botanists and others in achieving the 17 United Nations Sustainable Development Goals (SDGs, Q7649586) (https://www.un.org/sustainabledevelopment/, date last accessed, 15 May 2025), using botanical examples in the following themes: cultivation, conservation, and collaboration as a community (Knapp, 2024). This final theme—collaboration as a community—resonates strongly not only in botany (e.g. Antonelli *et al.*, 2024), but also in Wikidata. Bringing these two communities together to collaborate and share Linked Open Data benefits us all.

Richer collaboration efforts are needed by botanists to increase the amount as well as the quality of botany-related information in Wikidata, which can in turn reduce the curatorial workload and improve the data quality in our institutional databases (Groom *et al.*, 2020). Intensifying collaborations between the botanical and the Wikidata communities can assist with targeted improvements of data, e.g. in workshops or edit-a-thons focusing on specific topics related to botany, plants, agents active in botany or botanical collections, including herbaria, botanical gardens and others. WikiProjects (Q16695773) currently exist or can be created to facilitate collaboration efforts on improving knowledge for specific topics and provide an opportunity to invite other Wikimedians (and botanists) to get involved and contribute.

Wikidata can be used to support efforts towards improving knowledge equity and can assist with the democratization of knowledge. More collaboration is needed to correct biases in Wikidata, such as the gender content gap as well as race and citizenship biases (Shaik *et al.*, 2021; Zhang and Terveen 2021; Centelles and Ferran-Ferrer, 2024). Collaboration is also needed to improve the accessibility of data about botanical institutions and individual botanists from the Global South (Auge *et al.*, 2024) as well as marginalized communities or collections from colonial contexts (Kaiser *et al.*, 2023; Kaiser and von Mering, 2024). Contributions from botanists based in different countries can assist with these efforts. For example, the research undertaken on plant genera named in honour of women and mythical female beings (von Mering *et al.*, 2023*a*) was completed by eight co-authors from four countries.

The extended community of people working in botanical (or other natural history) collections and in the field of biodiversity data or biodiversity informatics collectively has a lot of knowledge and experience in the modelling of biodiversity-related information. Bringing together this community with Wikimedians and especially Wikidata editors helps to establish community-agreed data models in Wikidata that in turn facilitate the addition and enrichment of botany-related data. Such exchange and collaboration can also involve TDWG, the organization dedicated to developing biodiversity information standards. For example, the TDWG Task Group Modelling Research Expeditions comprises botanists, collections staff, data experts and Wikidata editors.

It is a fruitful collaboration for botanists to provide the broader Wikidata community with recommendations on how to model and link research-related data (including botanical expeditions) in Wikidata. An example of a successful collaboration is the creation and use of the property *collection items at* (P11146). In Wikidata, both the ontology of the database—including its properties, as a *Wikidata property proposal* (Q114746893)—as well as the data itself are all editable by the community. This property was therefore proposed to the Wikidata community for creation after consultations with biodiversity data experts and colleagues from different natural history collections (https://www.wikidata.org/wiki/Wikidata:Property_proposal/collection_items_at, date last accessed, 15 May 2025). It was accepted by the Wikidata community and is now widely used in Wikidata to link people or expeditions with institutions or collections housing specimens gathered by these people or during these collecting events.

The modelling of taxonomic data continues to develop in Wikidata with the recent creation of such properties as *taxon synonym of* (P12763) and *basionym of* (P12766) assisting with the enriching of botanical-related *items* (Fig. 9). Collaborations can ensure these properties are used more frequently and consistently, leading to richer botanical-related taxonomic data being integrated into Wikidata. Such data models also facilitate the better integration of and linking to large botanical databases such as IPNI and WFO.

In times of machine learning and AI, availability and easy accessibility of high-quality open data are important as free training data and for the verification of information. Data in open and community-curated platforms such as Wikidata are becoming more and more important when thinking about knowledge equity (Schuster, 2024). A project by Wikimedia Deutschland (WMDE) aims to facilitate the use of open data from Wikidata by open-source initiatives and non-profit AI projects (Schuster, 2024). Wikidata is used for fact-checking and verification, e.g. by combining structured data in its knowledge graph with large language models (LLMs; e.g. Thornton *et al.*, 2022). LLMs can also help to accelerate access to scholarly data hidden in unstructured datasets and make them more sustainably accessible via Wikidata (e.g. Mihindukulasooriya *et al.*, 2024).

### Getting started in Wikidata for botanists and botanical institutions

As an open and collaborative community, Wikidata empowers anyone to contribute and help to fill gaps, e.g. by adding information about botanists, botanical institutions, botanical taxa, etc. This can be the addition of small but important pieces of information such as a year of birth or death of a botanist, linking to the institution that holds archival material to the relevant botanist, adding the geolocation information for a botanical institute, and so on. Contributions can also be the batch or bulk upload of larger curated datasets, e.g. data about a botanical collection and its related collection agents. Every editor’s level of engagement with Wikidata varies over time, but every contribution, whether small or large, improves Wikidata.

As an individual, it is relatively easy to start manually editing Wikidata by making use of the many training materials available in multiple languages, including online tutorials (e.g. https://www.learnwikidata.net/, date last accessed, 15 May 2025), guidelines (Bauer *et al.*, 2022), publications (e.g. Shafee *et al.*, 2023; Leachman and Mabry, 2024) and resources available on Wikidata itself (e.g. https://www.wikidata.org/wiki/Wikidata:Tours and https://www.wikidata.org/wiki/Wikidata:WikiProject_IBC_2024/Resources, date last accessed, 15 May 2025).

Individuals are also able to edit in bulk using tools such as QuickStatements or OpenRefine. They are also encouraged to reach out to the Wikidata editing community to collaborate on botany-related projects to improve Wikidata. Connecting with fellow editors can take the form of attending in person or virtual Wiki meetups found locally, signing up to and engaging with relevant WikiProjects (such as https://www.wikidata.org/wiki/Wikidata:WikiProject_Botany, https://www.wikidata.org/wiki/Wikidata:WikiProject_Taxonomy and https://www.wikidata.org/wiki/Wikidata:WikiProject_Biodiversity, date last accessed, 15 May 2025) or other collaborations, such as Wikidata edit-a-thons, hackathons or conferences.

Botany institutions are encouraged to add their data to Wikidata (e.g. Lemus-Rojas and Lee, 2020). Institutions can also collaborate with the Wikidata community to create Wikidata properties for any appropriate institutional identifiers in order to link to the institution’s data in Wikidata (see Wikidata search https://bit.ly/3P1GqCy, date last accessed, 15 May 2025). Institutions can make use of the many tools available (such as Mix’n’match) to assist in the ingesting and linking of an institution’s data.

### Roundtripping data via Wikidata

Institutions are also encouraged to roundtrip data from Wikidata. *Data roundtripping* (Q108684293) is the process of using, reusing, enriching and updating data from one database and then integrating that same data into another. By adding links to appropriate Wikidata *items* in their collection management systems and on their websites, botany institutions can accelerate the accessibility and impact of their botany-related data. Should they be willing to do so, institutions are also able to ingest any needed information sourced from these Wikidata *items* into their own websites and collection management systems (e.g. BHL; Dearborn and Leachman, 2023).

To support organizations in engaging with Wikidata, the Wikidata page Linked Open Data workflow (https://www.wikidata.org/wiki/Wikidata:Linked_open_data_workflow, date last accessed, 15 May 2025) gives an overview of the steps that can be taken by the institution, and lists tools and scripts that can be used in the process of preparing, reconciling, ingesting, analysing, reusing and reporting of the impact of the data. It focuses on tools that are especially useful to galleries, libraries, archives and museums (GLAM institutions, Q1030034; see also https://www.wikidata.org/wiki/Special:MyLanguage/Wikidata:GLAM, date last accessed, 15 May 2025).

### How to integrate Wikidata in the classroom

Wikidata and Bionomia can also be easily integrated into university courses and can be used to support research undertaken by master’s and doctoral students. There are many online tutorials and other open educational resources (OER, Q116781) available that support teaching about these tools. Two modules that were recently developed as part of a course-based research experience (CURE) focus on Wikidata and Bionomia, respectively (Leachman and Mabry, 2024; Mabry *et al.*, 2024).

### Some ethical and legal considerations when contributing to Wikidata

Contributors to Wikidata should consider both ethical and legal guidelines and obligations when contributing to Wikidata. For example, when adding data to Wikidata, it is good practice to add authoritative, accessible and relevant references to all statements to allow any user to verify the information underlying the respective statements (Wikidata, 2025*b*). There are several initiatives within the Wikidata community to increase the number and quality of references on Wikidata statements, which would strengthen their validity, verifiability and reliability, and that of Wikidata as a whole (e.g. Piscopo *et al.*, 2017; Curotto and Hogan, 2020; Bauer *et al.*, 2022). When dealing with Wikidata *items* for under-represented botanists, it is often a challenge to find any references. In these cases, we believe it is better to provide unreferenced data or non-authoritative sources, as including these helps to surface additional information about under-represented people.

Copyright licensing must also be taken into account. Since Wikidata adheres to a CC0 licence, this makes it difficult to incorporate data with incompatible licensing. Despite these limitations, Wikidata remains a valuable tool for scientific research.

Wikidata editors should also follow the guidance given by Wikidata on editing *items* about themselves, or someone they know or represent (https://www.wikidata.org/wiki/Wikidata:Autobiography).

Contributors to Wikidata should consider the ethical and legal obligations that exist to protect a living person’s right to privacy (Lindsey *et al.*, 2022; Weathington and Brubaker, 2023). Wikidata has an agreed policy relating to living people which should also guide contributions (https://www.wikidata.org/wiki/Wikidata:Living_people).

In addition, Wikidata editors should consider appropriate CARE principles (Collective benefit, Authority to control, Responsibility, and Ethics; https://www.gida-global.org/care, date last accessed, 15 May 2025) (Jennings *et al.*, 2003) when adding or linking indigenous people’s data or data sourced from indigenous communities. Although the authors of this paper have emphasized how Wikidata can facilitate the reuse of botany-related data, we believe this should not be at the expense of the rights indigenous people have to control data sourced from their communities, nor should this restrict the application and use of indigenous data and knowledge for the benefit of indigenous communities.

## CONCLUSIONS

We argue that Wikidata is a key and timely platform that can and should support botany, botanists and botanical institutions, and the botany community’s collective goals and strategic actions. To achieve this, we botanists need to actively participate in Wikidata digital outreach. By steadily, deliberately and collaboratively improving the quality, quantity and linkages of botany-related data in Wikidata, botanists can—via individual and collective actions—harness the power of Linked Open Data to answer important queries in the field, support the Madrid Declaration strategic actions (Table 1) and ultimately make botany-related information more FAIR and equitable. Collaboration is key in botany and Wikidata, and sharing Linked Open Data benefits us all.
